# Characterization of Sheared Edges in Warm Blanking of Magnesium Alloy AZ31B

**DOI:** 10.3390/ma12071023

**Published:** 2019-03-28

**Authors:** Piemaan Fazily, Jaehyeong Yu, Chang-Whan Lee

**Affiliations:** CAE for advanced Materials (CAEM) Laboratory, Department of Mechanical Design and Manufacturing Engineering, Seoul National University of Science and Technology, Seoul 01811, Korea; piemaanfazily@seoultech.ac.kr (P.F.); jhyu9109@seoultech.ac.kr (J.Y.)

**Keywords:** blanking process, magnesium alloy AZ31B, sheared edge quality, shear affected zone, edge strain hardening index, microstructure

## Abstract

This research aims to characterize damage at the sheared edge caused by the blanking operation of magnesium alloy AZ31B sheets. Shearing tests were carried out on an in-house blanking die-set and mechanical press (universal testing machine) by varying punch–die clearance and temperature. Edge damage was distinguished by the geometrical features of the sheared edge and by the distribution of the edge strain hardening (ESH) index. In this account, optical microscopy and scanning electron microscopy were applied to examine the characteristic dimensions of the sheared edge, fracture profile, and sheared edge quality, while the Vickers hardness test was applied to observe the surface micro-hardness in the shear zone (SZ) and the shear affected zone (SAZ). It was concluded that the blanking of magnesium alloy sheets at room temperature results in sheared edge defects, due to premature fracture, referred to here as micro-cracks, loose particles, and a jagged-plus-curved fracture profile. However, such deformities were completely suppressed with the rise in temperature. In addition, based on optical morphology, micro-hardness tests, and microstructure evolution, the recommendation regarding blanking temperature for the magnesium alloy AZ31B has was proposed.

## 1. Introduction

Blanking operations are material shearing processes. They involve elastic and plastic deformations and a fracture of sheet-metal between a punch and a die. The shearing phenomena may introduce shear-induced damage at the sheared edges and strain hardening at its localities. Therefore, a conventional blanking process may result in inadequate quality blanks. Konieczny et al. [1] evaluated the influence of sheared edge quality and observed that blanking incurred reduced formability in hole expansion tests, as compared to laser cutting. Golovashchenko et al. [2] examined the effect of trimmed edges and reported reduced formability in stretch flanging operations. Furthermore, Shih et al. [3] reported that defects in the blanked edge could lead to premature edge fracture in the subsequent forming processes. Therefore, in order to improve the quality of the sheared edge, the sheared specimen may require additional post-processing steps [4], i.e., deburring [5]. 

A commercial magnesium alloy (AZ31B) sheet, due to its favorable mechanical properties (ultralight, damping capacity, and yield strength), is comparable to aluminum alloys, often used in lightweight automotive applications [6]. However, shearing of AZ31 sheets at ambient temperature results in an unusual zig-zag fracture profile at the sheared edge that can restrict further forming operations [7]. This behavior can be attributed to the hexagonal close-packed (hcp) crystalline structure of AZ31 sheets that leads to its strong in-plane anisotropy at ambient temperatures [8]. Furthermore, plastic deformation at ambient temperature is confined and mechanical twining is the dominant deformation mechanism [9]. However, across the temperature range 150–250 °C, non-basal deformation that significantly improves material ductility is activated [10]. In this regard, Nürnberg et al. [11] examined the blanking of magnesium alloy sheets and observed an improved sheared edge quality through the texture modification of the AZ31 sheet. Similarly, Scintilla et al. [12] investigated fiber laser cutting of AZ31 sheets and determined the ample cutting-edge quality of the workpiece.

The literature review demonstrates the effects of blanking process parameters on the formation of the sheared edge and the extent of strain hardening in the shear affected zone (SAZ). Accordingly, the characteristic dimensions of the sheared edge and strain hardening at its vicinities are influenced by the variation in process parameters, such as punch–die clearance [13,14], shear angle [15,16], punch–die radius [17], punch speed [18], and temperature [19,20]. Meanwhile, the strain hardening in the SAZ can be identified either by micro-hardness measurements through the material thickness using nanoindentations [21,22] or by optical microscopic measurements of the deformation angle, with respect to metal flow lines [23].

The primary objective of this work is to conduct an experimental study to improve the sheared edge quality and provide recommendations regarding process variables in the blanking operation of magnesium alloy AZ31B. Shearing tests were carried out on the magnesium alloy workpieces and the damage that may arise in the respective sheared edges were characterized based on the assessment methods reported in Section 2.1. The assessments account for the effects of variation in process parameters, including punch–die clearance and temperature, on the quality of the sheared edge. Furthermore, microstructure observations provided conclusive insights into the quality of the blanked edge.

## 2. Materials and Methods

### 2.1. Edge Damage Assessments

**Figure 1:**
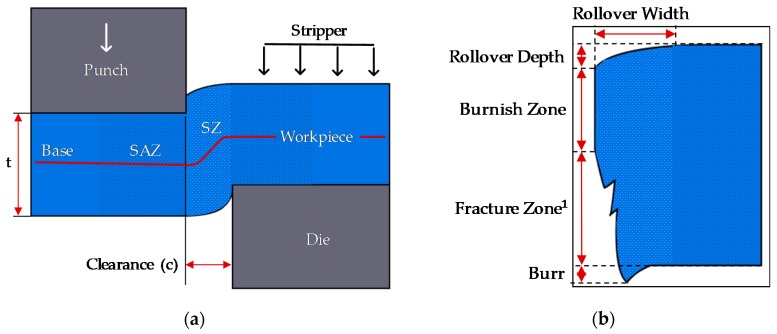
(**a**) Schematic of the blanking process; (**b**) illustration of the sheared edge profile, consisting of rollover region, burnish zone, fracture zone, and burr. ^1^ Fracture zone value excluding burr.

Primarily the sheared edge formed through the blanking process is distinguished by regions known as the rollover depth, rollover width, burnish zone, fracture zone, and burr, as illustrated in Figure 1. Their characteristic dimensions are measured by using either optical or scanning electron microscopy TESCAN VEGA3 (Seoul, Korea). The assessment reveals the morphology of the fracture profile, in correlation with the sheared edge quality.

Alternatively, micro-hardness contours of the blanks can provide quantitative insights into strain hardening and thermal effects in the region. 

Chiriac et al. [24] proposed an edge strain hardening index as:

(1) $$\mathrm{ESH}\;[\%]\;=\;\frac{Hv-Hv_{base}}{Hv_{base}},$$

where Hv represents micro-hardness in the shear zone and shear affected zone and $Hv_{base}$ is the micro-hardness in the base substrate. 

The value of the edge strain hardening (ESH) index in Equation (1) is obtained through the Vickers hardness test.

### 2.2. Experimental Setup

**Figure 2:**
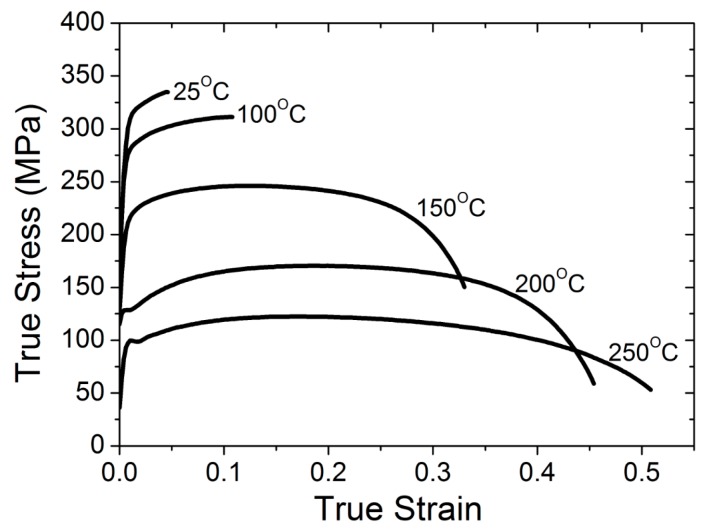
Stress–strain curves of the uniaxial tensile tests at various temperatures for the magnesium alloy (AZ31B), along 0° to the rolling direction and 0.16/s strain rate, obtained from the NUMISHEET 2011 benchmark study (BM 2) [25].

The blanking workpieces were taken transverse to the rolling direction of the magnesium alloy (AZ31B) sheet. The final workpiece had a rectangular shape and a thickness of 1 mm. The chemical composition of the magnesium alloy (AZ31B) is shown in Table 1. The material data for the magnesium alloy at temperatures of 25 °C, 100 °C, 150 °C, 200 °C, and 250 °C is shown in Figure 2.

**Figure 3:**
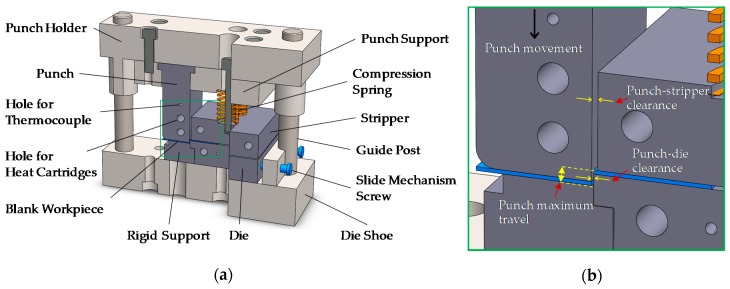
(**a**) Main components of the blanking device (sectional view); (**b**) magnified illustration of the selected blanking parameters.

Shearing tests were employed on an in-house blanking device and a 100 kN mechanical press (UTM). The sectional view of the blanking device used for the experiments is shown in Figure 3a,b. Both the punch and die were fabricated with Cr-alloy tool Steel SDK61. For the efficient stripping of blank from the punch, the blank holder was spring-loaded. The punch–die clearance was controlled by the side-screw implement that enabled horizontal movement of the die. In addition, punch, die, and blank holder were pre-drilled and reamed to aid the insertions of heat cartridges.

### 2.3. Experimental Procedure

During the blanking experiment, the punch holder was connected directly with the crosshead of the universal testing machine. Hence, the load-displacement response measured was adjusted to segregate spring reaction and the blanking force. The blanking workpiece was heated and constant temperatures across the tooling setup were maintained by the cartridge heater, using the PID temperature controller. Clearance to thickness ratios was recalibrated using an appropriate thickness gauge to account for thermal expansion during the high-temperature arrangements. Optical microscopy and scanning electron microscopy were applied to examine the characteristic dimensions of the sheared edge, fracture profile, and sheared edge quality. Summary of the punch–die clearance, temperature, and other process parameters is shown in Table 2.

The resulting blanked specimen was sectioned and cold-mounted, using a cold-setting resin. The mounted samples were thereby ground and mechanically polished, using SiC papers and alumina powder, respectively. For visualizing grain deformations, samples were chemically etched with the solution (4.2 g picric acid, 70 mL ethanol, 10 mL distilled water, and 75 mL acetic acid) for about 5 s. Optical microscopy and scanning electron microscopy were applied to obtain the micrographs of the mounted samples.

Micro-hardness indents were conducted using the micro-hardness tester. The tests were employed across the thickness of the material by applying a test load of 100 gf for a dwell time of 5 s. Vickers hardness along the shear zone (SZ), SAZ, and the base substrate were measured at various positions over the domain of the mounted sample.

## 3. Results and Discussion

### 3.1. Geometrical Features of the Sheared Edge

**Figure 4:**
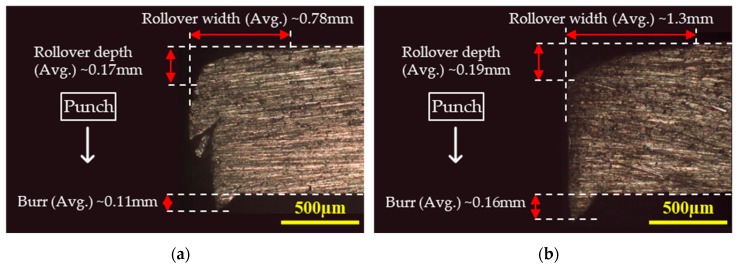
Side-view (OM) of the blanked edge obtained from experiments for 20% clearance at (**a**) 25 °C, indicating primary jagged fracture geometry and secondary curved fracture profile, and (**b**) 250 °C, indicating smooth sheared edge.

**Figure 5:**
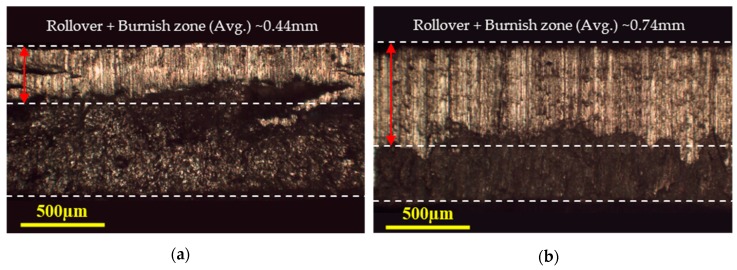
Front view (OM) of the blanked edge obtained from experiments for 20% clearance at (**a**) 25 °C, indicating micro-cracks in the burnish zone and loose fragments, and (**b**) 250 °C, indicating positively improved sheared edge.

The sheared edges were examined under optical microscopy (OM) and scanning electron microscopy (SEM) through the following four steps:

1. Rollover depth, rollover width, and burr heights for each blank were measured and averaged from its two side-view OM images. The side-view morphology corresponding to 20% clearance (expressed as the percentage ratio of punch–die clearance to the workpiece thickness) and temperatures of 25 °C and 250 °C are shown in Figure 4a,b respectively.
2. Burnish zone and fracture zones were quantified from the normal morphologies of the sheared edges. The dimensions were measured at various positions and averaged to account for variations. The front-view morphology corresponding to 20% clearance and temperatures of 25 °C and 250 °C are shown in Figure 5a,b respectively.
3. Detailed features of the fracture profile were obtained from the scanning electron microscopy in an oriented view of the sheared edge.
4. To analyze sheared edge quality, the blanks were further examined under SEM for obtaining fracture morphologies.

**Figure 6:**
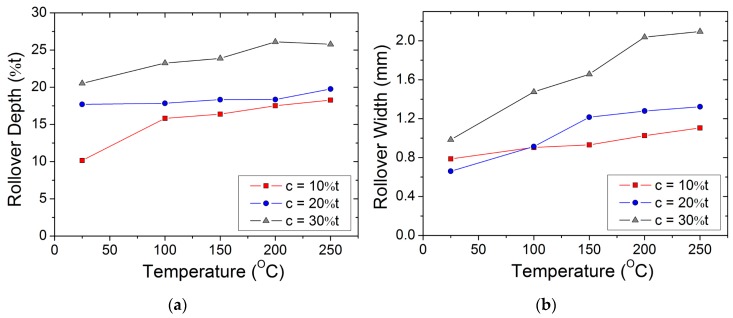
(**a**) Variation of the rollover depth (expressed as a percentage of the workpiece thickness) with temperature for various punch–die clearances; (**b**) variation of the rollover width with temperature for various punch–die clearances.

The rollover depth was measured as the vertical distance between the upper plane of the workpiece and the onset of the burnish zone. It was observed that the rollover depth increases with the increase in temperature and punch–die clearance. The rollover depth at 10% clearance increased from approximately 0.1 mm at ambient temperature to approximately 0.18 mm at 250 °C. Similarly, at 30% clearance and 250 °C, the rollover depth increased up to approximately 0.25 mm. Increase in temperature (increase ductility) and clearance (wider processing zone) delayed punch penetration of the workpiece, hence, significant rollover region was observed. Variations in rollover depth, along with temperature, are shown in Figure 6a. 

The rollover width defines the extent of bending deflection in the blank caused by the punch contact. It was measured as the horizontal distance between the beginning of the bent and the burnish zone. It is observed that the rollover width increases with the increase in temperature and clearance. For instance, the rollover width at 10% clearance increased from approximately 0.78 mm at ambient temperature to approximately 1.3 mm at 250 °C. The rollover width against the temperature plot is shown in Figure 6b.

**Figure 7:**
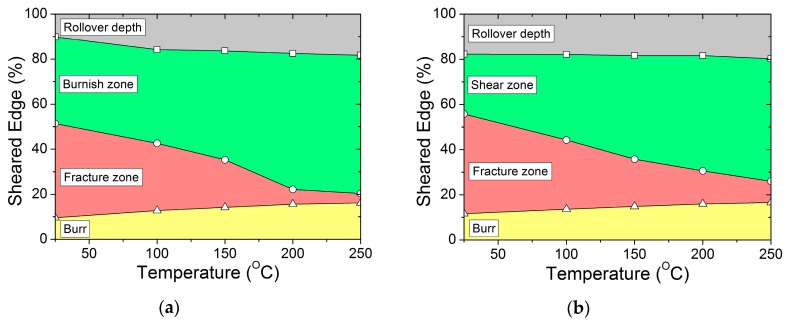
Relationship between characteristic dimensions of sheared edge (%) and temperature at (**a**) 10% clearance and (**b**) 20% clearance.

**Figure 8:**
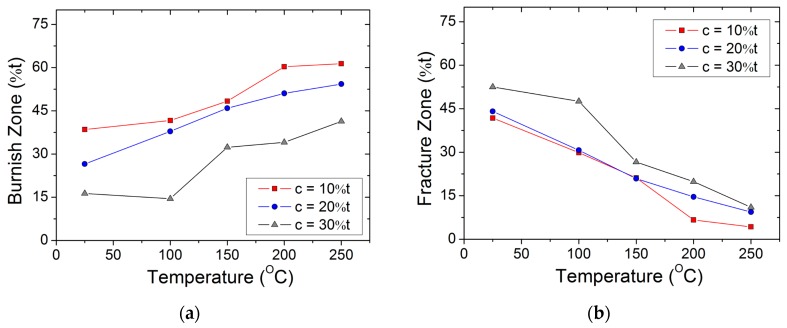
(**a**) Variation of the burnish zone (expressed as a percentage of the workpiece thickness) with temperature for various punch–die clearances; (**b**) variation of the fracture zone (expressed as a percentage of the workpiece thickness) with temperature for various punch–die clearances.

The burnish zone features a set of vertical marks on the sheared edge, caused by the compression forces between punch and workpiece. It was observed that the burnish zone increases with the increase in temperature and decreases with the increase in clearance. At ambient temperature, additional micro-cracks are observed in the burnish zone, parallel to the sheet plane. Further punch penetration results in the fragment (loose particles) chipping away and then initiating the main crack to propagate through the material thickness. However, with an increase in temperature (or to an increase in ductility), no micro-cracks or loose particles were observed in the burnish zone. In addition, at a lower clearance regime (or an increase in hydrostatic stresses), the burnish zone may also stretch up to the lower plane of the sheet. The relationship between the sheared edge (%) of the characteristic region at 10 t and 20 t clearances are illustrated in Figure 7a,b, respectively. Variations in burnish zone height, along with temperature, are shown in Figure 8a.

**Figure 9:**
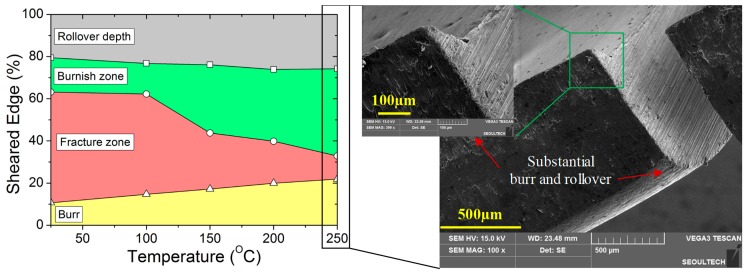
Relationship between characteristic dimensions of sheared edge (%) and temperature at 30% clearance, indicating a substantial rollover region and burr feature.

The fracture zone features rough surfaces on the sheared edge, caused by the material splitting. In this paper, fracture zone value was measured as the vertical distance up to the lower plane of the workpiece, excluding burr. It was observed that the fracture zone is reciprocal to the burnish zone, hence, its value decreases with the increase in temperature. Variations in fracture zone height, along with temperature, are shown in Figure 8b. However, the fracture zone shows an increasing trend with the increase in clearance and, therefore, even at 250 °C, the fracture zone value is observed to increase from approximately 0.043 mm at 10% clearance to approximately 0.11 mm at 30% clearance. Furthermore, the increase in temperature and clearance also resulted in a substantial rollover region and burr in the blank, as illustrated in Figure 9.

**Figure 10:**
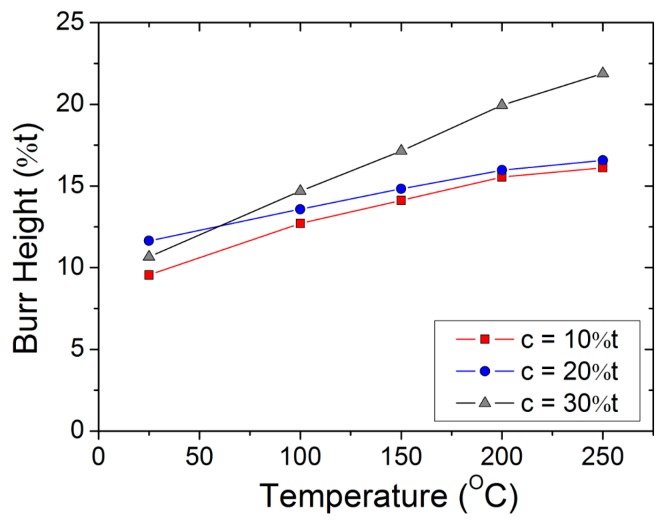
Relationship between burr height (expressed as a percentage of the workpiece thickness) and heating temperature for various punch–die clearances.

The burr height was measured as the vertical distance between the lower plane of the workpiece and the end of the metal fragment. It was observed that the burr increases with the increase in temperature and clearance. The burr height at 10% clearance increased from approximately 0.09 mm at ambient temperature to approximately 0.16 mm at 250 °C. Similarly, at 30% clearance and 250 °C, the rollover depth increased up to approximately 0.21 mm. Variations in burr height, along with temperature, are shown in Figure 10.

**Figure 11:**
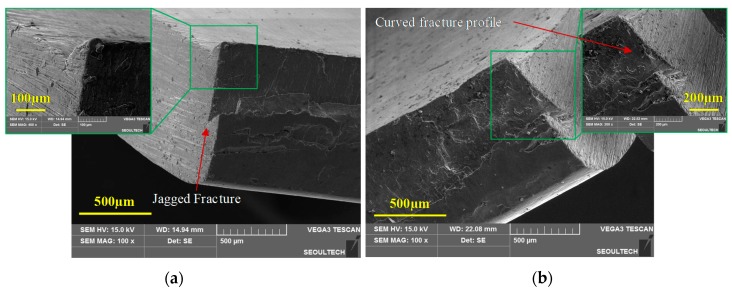
Oriented view (SEM) of the blanked edge obtained from experiments for 20% clearance at 25 °C: (**a**) Side 1, illustrating primary jagged fracture geometry; (**b**) Side 2, illustrating secondary curved fracture profile.

**Figure 12:**
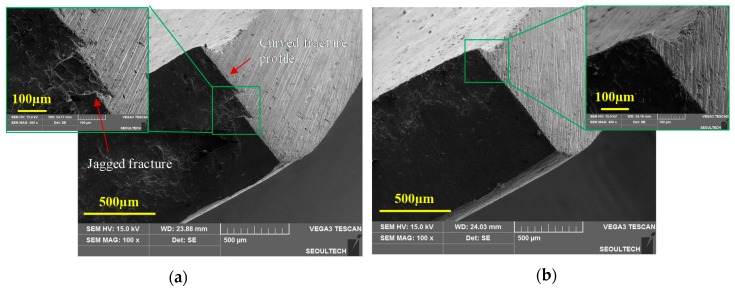
Oriented view (SEM) of the blanked edge obtained from experiments for 20% clearance at (**a**) 100 °C, illustrating jagged-plus-curved fracture profile, and (**b**) 250 °C, illustrating smooth sheared edge.

Blanking of a magnesium alloy sheet at room temperature results in an unstable propagation of cracks after its initiation across the material thickness. The instability in crack evolution can pave additional cracks to take multiple paths and lead to micro-cracks. The random crack pattern influences the sheared edge geometry to develop a jagged fracture profile, evident from the previous sectional images. This behavior can be attributed to the strong in-plane anisotropy of AZ31B sheets along the normal direction (ND) at temperatures below 150 °C. In addition, the optical micrographs also revealed the formation of a secondary curved fracture profile in the sheared edges of 25 °C and 100 °C. Representation of scanning electron microscopy at 25 °C, corresponding to 20% clearance, is illustrated in Figure 11a,b. The morphologies are in an oriented view of the two sides of the same blank. Similarly, SEM micrographs at 100 °C and 20% clearance show identical defects of a jagged-plus-curved fracture profile, as shown in Figure 12a. This unusual zig-zag fracture profile at the sheared edge may lead to premature failures during further forming operations. However, the problem of the jagged fracture as well as curved fracture profile was completely suppressed at blanking temperatures of 150 °C and beyond. The SEM image of the fracture profile at 250 °C, corresponding to 20% clearance, is shown in Figure 12b.

The quality of the sheared edge formed by the blanking of magnesium alloy sheet has a positive correlation with temperature. Due to unstable crack propagation at ambient temperatures, additional micro-cracks are generated in the burnish zone, parallel to the sheet plane. This can lead to the formation of fragmented particles chipping away at the sheared edge. Scanning electron microscopy observations of the sheared edge indicated defects like micro-cracks and loose particles.

**Figure 13:**
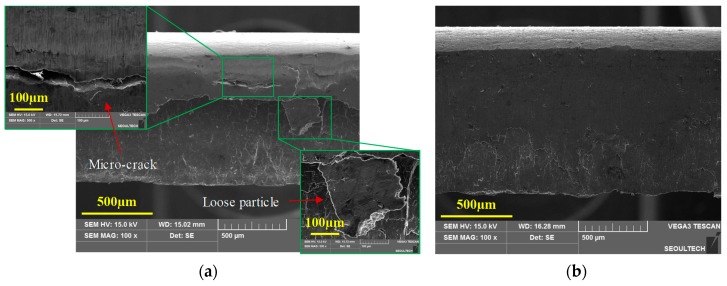
Front view (SEM) of the blanked edge obtained from experiments for 20% clearance at (**a**) 25 °C, illustrating micro-crack in the burnish zone and loose particles, and (**b**) 250 °C, illustrating positively improved sheared edge.

**Figure 14:**
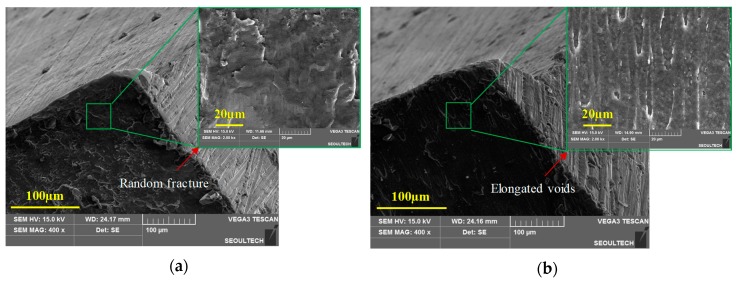
SEM image (2000×) of the fracture surface for 20% clearance at (**a**) 25 °C, illustrating highly random fracture surface with no visible dimples, and (**b**) 250 °C, illustrating elongated dimples in the fracture surface.

The normal morphology of the sheared edge at 25 °C and 20% clearance is shown in Figure 13a. These deformities at the sheared edge may lead to edge fracture during subsequent operations. However, the sheared edge quality of the magnesium alloy blanks is effectively improved with the increase in temperature. The normal SEM morphology of the sheared edge at 250 °C, corresponding to 20% clearance, is shown in the Figure 13b. Furthermore, normal SEM morphologies of the fracture surface at higher magnifications provided conclusive insights into the fracture mechanism of AZ31B sheet. It can be observed that the fracture surface of the blanks obtained at ambient temperature is highly random with no visible dimples. Representation of SEM micrographs (2000× magnification) at 25 °C, corresponding to 20% clearance, is illustrated in Figure 14a. However, at temperatures of 200 °C and beyond, elongated dimples in the fracture surface are observed, indicating a ductile fracture. The SEM image (2000×) of the fracture surface at 250 °C and 20% clearance is illustrated in the Figure 14b.

**Figure 15:**
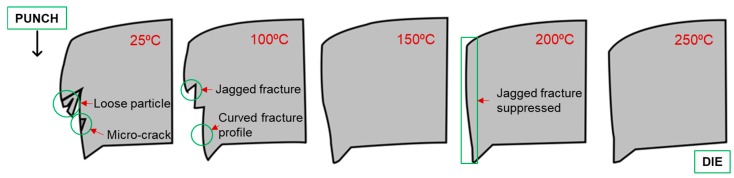
Schematic comparison of sheared edge profiles at temperatures ranging from 25–250 °C and punch–die clearance of 20%.

In conclusion, blanking operation of magnesium alloy (AZ31B) at 25 °C and 100 °C resulted in sheared edge defects, such as loose particles, microcracks, jagged fracture profile, and curved fracture profile. The schematic of the sheared edge profiles at 20% clearance and various temperatures is shown in Figure 15. These deformities may severely affect the formability of the consequent forming operations. On the contrary, blanking of magnesium alloy in warm forming conditions completely suppressed such defects. However, the increase in temperature also increased the rollover region and burr, which are also important quality benchmarks. Therefore, the blanks were further examined for strain hardening or increase/decrease in hardness in the following section.

### 3.2. Distribution of Edge Strain Hardening Index

The mounted samples of the sectioned blanks were subjected to nanoindentations, using a micro-hardness tester. The value of the ESH index in Equation (1) was calculated and plotted against distance within the SZ and the SAZ.

**Figure 16:**
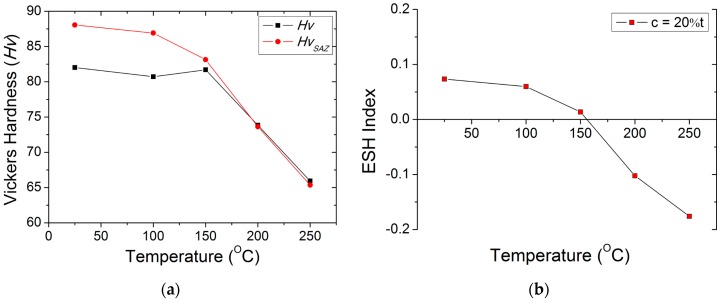
(**a**) Plot between Vickers hardness and heating temperature for the punch–die clearance of 20%; (**b**) plot between edge strain hardening (ESH) index and heating temperature for the punch–die clearance of 20%.

It can be observed that the hardness measurements are sensitive to the increase in temperature. One batch of samples with individual clearance was chosen to quantify the effect of temperature. Vickers hardness was measured as an average value of five measurements, performed separately near shear zone vicinities and the base substrate. It was observed that the material hardness decreases with the increase in temperature for both regions. The material hardness at the base substrate decreased from 82 μH_V_ at the ambient temperature to 69 μH_V_ at 250 °C, while material hardness near shear band vicinities decreased from 88 μH_V_ at the ambient temperature to 68 μH_V_ at 250 °C. The Vickers hardness test against the temperature plot is shown in Figure 16a. The hardness value in the base substrate at 25 °C was used as a reference hardness value ($Hv_{base}$) for the subsequent ESH index calculations. Variations in the ESH index, along with temperature, are shown in Figure 16b. The positive ESH index value indicates strain hardening at the shear zone vicinities, which decreases with the increase in temperature. After 150 °C however, the thermal effects in the blanks overcome strain hardening, hence, negative ESH index values are observed. 

**Figure 17:**
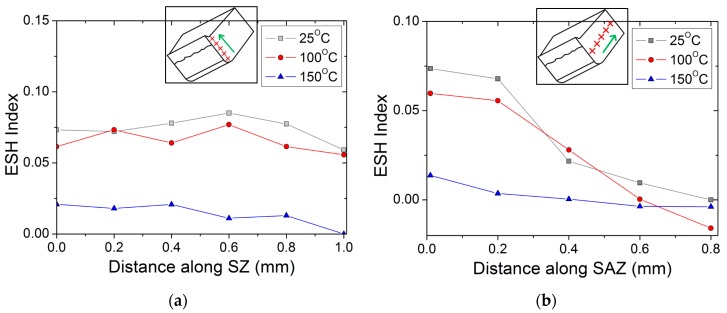
(**a**) Distribution of ESH index along shear zone; (**b**) distribution of ESH index along shear affected zone.

The distribution of the ESH index along the shear affected zone and shear zone quantifies the extent of strain hardening and damage caused by the blanking process. Micro-hardness was measured across the SZ, from the rollover region to the burr feature. The distribution of the ESH index is then calculated using the reference hardness value ($Hv_{base}$) and plotted against temperature in Figure 17a. It can be observed that the distribution of the ESH index along the sheared edge at 150 °C is approximately 80% lower than that of 25 °C. 

Micro-hardness was measured across the SAZ, from the sheared edge to the base substrate. The distribution of the ESH index is then calculated using the reference hardness value ($Hv_{base}$) and plotted against temperature in Figure 17b. It was observed that the ESH index value decreases from the sheared edge to the interior. For instance, the shear affected zone at 25 °C is approximately 0.8 mm while at 150 °C, it is approximately 0.4 mm. However, at temperature 150 °C, the gradient of the ESH index is relatively smaller. Therefore, the positive gradient of the ESH index virtually disappears in the SAZ with the increase in temperature.

### 3.3. Microstructure Observations

**Figure 18:**
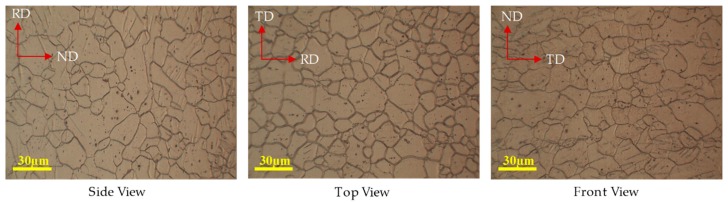
Initial microstructure of as-received magnesium alloy sheet AZ31B.

The etched samples of the sectioned blanks were examined under the optical microscopy for obtaining texture measurements. Grain sizes were measured by the linear intercept method, corresponding to ASTM standard E112 using OM. The initial microstructure of as-received magnesium alloy in the rolling (RD), transverse (RD), and normal (ND) direction is shown in Figure 18. The average grain size is 30 μm in the thickness direction.

**Figure 19:**
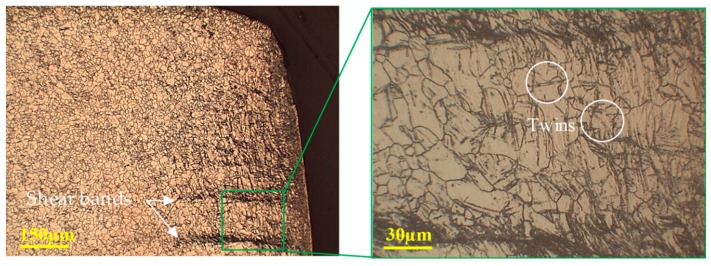
Optical microstructure of the shear zone at 25 °C and 20% clearance, illustrating shear bands and twinning at the sheared edge.

**Figure 20:**
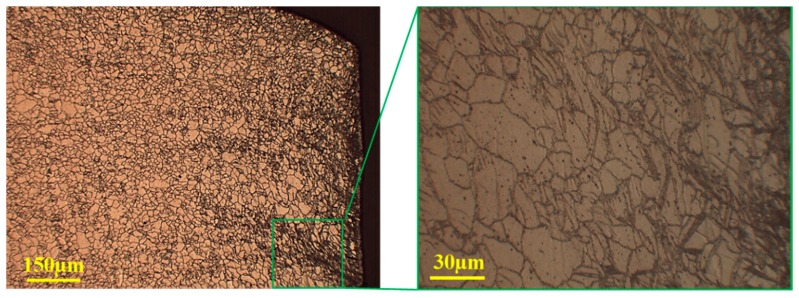
Optical microstructure of the shear zone at 150 °C and 20% clearance.

It can be observed that mechanical twining dominated the plastic deformations at temperatures below 150 °C in the magnesium alloy sheet. The sheared edge obtained at 25 °C exhibits evidence of twins, as illustrated in Figure 19, which induces barriers to slip deformation (strain hardening). As a result, stress concentrations at the barriers work to form shear bands across the grain boundaries. Further deformation causes micro-cracks to develop within shear bands. The propagation of micro-cracks along the shear bands leads to the final fracture of the workpiece. At ambient temperature, the instability of crack evolution in magnesium alloy paves additional shear bands to take multiple paths and lead to a typical jagged fracture in the sheared edge. However, the fraction of twins decreases with increasing temperature, as illustrated in Figure 20, suppressing the jagged fracture in the sheared edge. 

**Figure 21:**
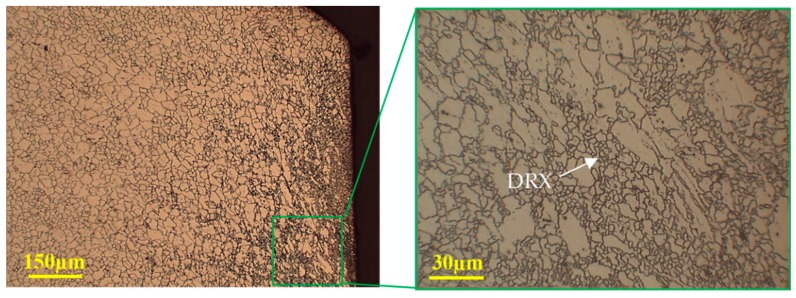
Optical microstructure of the shear zone at 250 °C and 20% clearance, illustrating dynamic recrystallization at the sheared edge.

Furthermore, the sheared edge obtained at 250 °C revealed dynamic recrystallization (DRX), with no evidence of twinning. The microstructure contains a bimodal distribution of recrystallized and elongated grains, as shown in Figure 21. The decrease in the ESH index with temperature in the previous section can also be attributed to the formation of DRX. Therefore, the microstructure evolution during shearing of magnesium alloy at various temperatures indicates a correlation to its mechanical behavior.

### 3.4. Maximum Blanking Force

**Figure 22:**
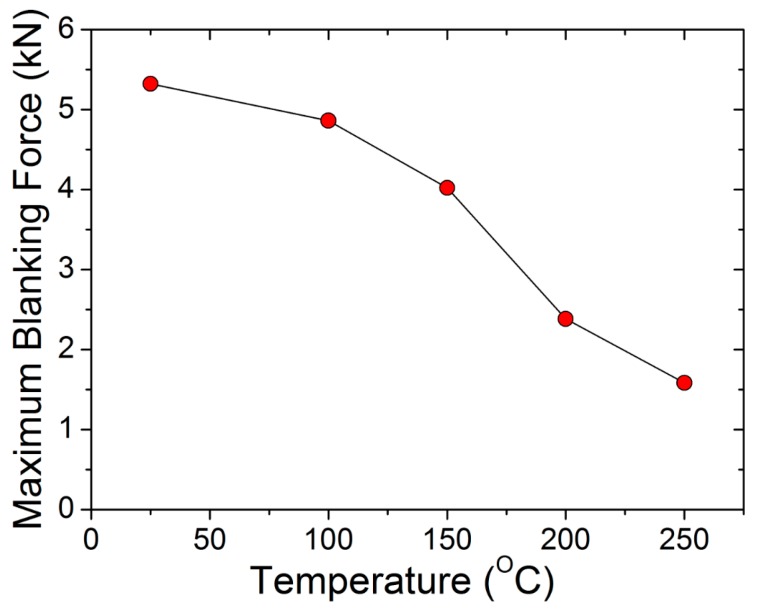
Relationship between maximum blanking force (as kN) and heating temperature for the punch–die clearance of 10%.

The load response measured by the load cell was a combination of spring reaction and blanking force. Hence, the reaction force was adjusted to segregate spring reaction and the blanking force. It was observed that, irrespective of the clearance value, the maximum blanking force decreases with the increase in temperature. The maximum blanking force at 10% clearance decreased from approximately 5.3 kN at ambient temperature to approximately 1.6 kN at 250 °C (30% of the cold blanking force). Variations in the maximum blanking force, along with temperature at 10% clearance, are shown in Figure 22.

## 4. Conclusions

The study aimed to characterize damage at the sheared edge and to improve sheared edge quality in the blanking operation of magnesium alloy AZ31B sheets. The edge damage was distinguished by morphologies (OM and SEM), micro-hardness tests, and microstructure observations. The effects of variation in process parameters, including punch–die clearance and temperature, on the quality of the sheared edge were also examined. It was observed that:

1. Based on OM and SEM morphologies, at temperatures of 25 °C and 100 °C, the sheared edge revealed defects, such as loose particles, microcracks, jagged fracture profile, and curved fracture profile. However, such deformities were completely suppressed at blanking temperatures of 150 °C and beyond. Furthermore:
  - Rollover depth and rollover width increase with the increase in temperature and clearance;
  - Burnish zone increases with increase in temperature and decreases with the increase in clearance;
  - Fracture zone is reciprocal to the burnish zone, hence its value decreases with the increase in temperature and increases with increase in clearance;
  - Burr height increases with increase in temperature and clearance.
2. Micro-hardness tests, at temperatures 25 °C and 100 °C, of the sheared zone vicinities revealed significant strain hardening. The phenomenon was demonstrated by the positive value of the edge strain hardening index, which decreases with an increase in temperature. Furthermore:
  - The distribution of the ESH index along the shear zone decreases with the increase in temperature;
  - The ESH index decreases from the sheared edge to the interior, indicating the extent of the SAZ. The positive gradient of the ESH index vanishes with the increase in temperature.
3. Microstructure observations of the sheared edge obtained at temperatures 25 °C and 100 °C revealed evident twins and shear bands. However, the fraction of twins decreases with the increase in temperature. The sheared edge obtained at 250 °C revealed dynamic recrystallization (DRX) with no evidence of twining.

Hence, warm blanking conditions have not only positively changed the sheared edge quality but also reduced the maximum blanking force. Based on the above observations, the temperature of 150 °C is recommended for the blanking operation of magnesium alloy AZ31B sheets.

## Figures and Tables

**Table 1:** The chemical composition of magnesium alloy AZ31B (wt %).

Mg	Al	Zn	Mn	Si	Fe	Cu	Ni
Bal.	3.15	1.02	0.54	0.0150	0.0032	0.0115	0.0028

**Table 2:** Tooling and experimental parameters.

	Parameters	Value
Tooling Setup	Punch–die material	Cr-alloy tool Steel SDK61
	Punch maximum travel	2 mm
	Shear angle	0°
	Compression spring	SWG20-40
	Initial workpiece dimensions	60 × 30 (mm × mm)
	Punch–die radius	0.05 mm (fictitious)
Experimental Setup	Workpiece thickness (t)	1 mm
	Punch–die clearance (c)	10, 20, 30 (% of thickness)
	Punch–stripper clearance	2.1, 2.2, 2.3 (mm)
	Punch speed (v)	2 mm/min
	Temperature (T)	25, 100, 150, 200, 250 (°C)
